# Text Messages in the Field of Mental Health: Rapid Review of the Reviews

**DOI:** 10.3389/fpsyt.2022.921982

**Published:** 2022-06-24

**Authors:** Reham Shalaby, Medard K. Adu, Hany M. El Gindi, Vincent I. O. Agyapong

**Affiliations:** ^1^Department of Psychiatry, Faculty of Medicine and Dentistry, University of Alberta, Edmonton, AB, Canada; ^2^Critical Care Medicine Department, King Abdul-Aziz Hospital, Jeddah, Saudi Arabia; ^3^Department of Psychiatry, Faculty of Medicine and Dentistry, Dalhousie University, Halifax, NS, Canada

**Keywords:** text messages, rapid review, mental health gap, e-mental health, mobile health

## Abstract

**Background:**

While mental health problems constitute a worldwide concern contributing to the global rates of morbidity and mortality, conventional mental healthcare services do not meet the current needs. Text messages (TM) represent a live model that incorporates technology into health services, spanning a large number of health conditions and playing different roles that may support the current healthcare system.

**Objective:**

To examine the TM services in the field of mental health, regarding their effectiveness, feasibility, acceptability, and economic evaluation in different contexts of mental health diagnoses and during critical times, when provided to individuals with mental health symptoms/disorders.

**Methods:**

This rapid review was conducted through an online search in PubMed, Embase, PsycINFO, and Medline databases. The review targeted the review studies which examined online or mobile addiction and mental health services, utilizing TM services. The search was run from the inception up to September 30, 2021.

**Results:**

Sixty review articles met the inclusion criteria and were included in this review. All reviews were published over the last decade. The results showed that people of a young age were fairly represented, and most reviews were run over substance use disorders (SUD), including Alcohol. Most reviews examined the effectiveness outcomes of the texting service, while to a lesser extent the acceptability and feasibility, among others. Texting services were reported as effective in psychotic disorders and SUD. However, the results related to depression and anxiety were mixed. Most reviews reported a considerably high risk of bias among their included studies. High satisfaction and acceptability of the texting services were reported for patients with various mental health conditions, including those with severe mental illness.

**Conclusions:**

This rapid review highlighted the applications, usability, benefits, and satisfaction with the TM in the field of mental health. For a higher quality of evidence, future studies should consider TM interventions in the contexts with mixed results or a dearth of literature, and during critical times, such as the COVID-19 pandemic. Policy- and decision-makers, therefore, need to further support text-based services with guided investments in interventions that were evidenced to be accepted, economic and feasible.

## Introduction

Mental health problems constitute a global concern and contribute significantly to the global rates of morbidity and mortality (1, 2). Mental health care services, however, do not meet current needs. Globally, about two-thirds of patients with mental illness never seek treatment (3). Similarly, in Europe, less than one-third of patients with psychiatric illnesses receive any treatment (4); in the USA, more than half of adults with mental illness do not receive treatment for their mental health conditions (5); and in Canada, more than 40% of Canadians who reported that they need help for mental health, reported their needs were partially met or fully unmet (6).

Although the World Health Organization (WHO) reported the availability of mental health services, such as medications (3), several other limitations have contributed to the aforementioned trends. For example, the system barriers represented in the lack of mental health professionals, including therapists in the remote and rural communities, in addition to the costly and resource-intensive nature of the conventional services (3, 5–8). Furthermore, the stigma and discrimination barriers related to the patients' own beliefs or to the social and professional circles surrounding them have significantly contributed to the limited accessibility to the mental health services (3, 5–8).

A large body of literature, therefore, suggested the high impact and effectiveness of the remotely delivered services that could be as effective in managing mental health conditions as compared to face-to-face services (6, 9). Skills acquired through self-directed learning from online resources and different technological platforms have been embraced as potential solutions to cross the gap between evidence-based therapies and community practice (10). Such services have been widely adopted in the healthcare system worldwide, and several technical terms becoming commonly used, such as telehealth or telepsychiatry, mHealth, eHealth, Internet interventions, mobile-based interventions, and user-led support (11–15).

Text messages represent a live model for incorporating technology into the health care system, spanning over a large scale of mental health conditions and through different roles. The majority of the current literature reports significant outcomes of these services within different contexts. For example, texting services are commonly used as a reminder of medical appointments (16) or to encourage adherence to prescribed medications (17). Texting or talking on the phone was a preferred mode of communication among adolescents compared to the face-to-face physicians' evaluation; more convenience with reduced anxiety and effective communication were among the reported reasons (18). Therefore, the growing spread of such wireless services in the health care system became more reasonable.

Texting service programs, such as Text2quit and Quit4baby, have been provided to people in the field of smoking, addiction, and well-being during their vulnerability with considerable success.

Text2quit is a smoking cessation text messaging program provided primarily for adults, while Quit4baby is a smoking cessation text messaging program for pregnant smokers that was adapted from Text2quit (19, 20). Like Text2quit, Quit4baby was based on the Social Cognitive Theory with messages aimed at improving self-efficacy for quitting. Participants received 1 to 5 text messages every day, with the highest dose of text messages sent on and around their quit date (20). Quit4baby was designed to serve as a potential adjunct service for Text4baby, an existing national texting program in the USA, provided for pregnant women that provides perinatal health information, and has enrolled almost 1,000,000 users since its launch in 2010 (20, 21). Text4baby is a theory-based mobile health program in which text messages are delivered to pregnant women and new mothers to improve their beliefs and behaviors of health care, and improve health status and clinical outcomes (22, 23). The mothers receive free text messages three times per week with interactive components and user feedback (21). Favorable responses to these programs have been reported. Participants to Text2quit and Quit4baby praised the content and the skills taught that helped them with positive ideas on quitting, to the extent that they may recommend it to a friend. Among reported outcomes for Text4baby, the positive belief changes among pregnant and new mothers concerned perinatal vitamin use, visiting a health care provider, and the risk of alcohol during pregnancy.

Text4Mood and Text4Support represent two other mobile text messaging programs to help people with mood disorders and other mental health conditions, including alcohol use disorder, respectively (24–27). Through Text4Mood service, subscribers were patients seeking psychological or counseling services for depression and anxiety who received one daily text message for six consecutive months (24). The messages were written by cognitive behavior therapists and counselors in partnership with mental health patients. Text4Support service, on the other hand, was delivered to patients with diverse mental health background. A bank of messages was generated and included different text message programs tailored for the following eight mental health domains: depression, anxiety, psychotic disorders, substance use disorders, bipolar disorder, adjustment disorders, attention-deficit or hyperactivity disorder, and general well-being (28). The subscribers received a single daily message for six consecutive months, which were based on cognitive behavioral therapy principles, and patients were enrolled to receive an assigned message bank based on their primary diagnosis.

The results were promising as the services reported improved depression symptoms on the BDI-II scores, the sense of better symptom control, and high satisfaction on several domains. Clinically, the service reported significant higher days to first drink in patients who received daily supportive text messages for 6 months, compared to those who did not.

The current availability of mobile phones has significantly contributed to the wide use of text message services (8, 29). Seven billion people, representing 95% of the population worldwide, live in an area covered by a mobile-cellular network (8). In Canada, the number of mobile internet users is 33.7 million in 2021 (30), representing around 90% of the population (88.1%). Additionally, by 2019, mobile technology expanded rapidly and the global penetration rate was 96%, with more than 8 billion mobile phone subscriptions worldwide, along with the global widespread wireless network (31).

During crises times, such as COVID-19 pandemic, restrictions and limited activities were enforced in many jurisdictions. Self-isolation, quarantine, and physical distance rules became the new normal during this hard time that added to the distress associated with the pandemic and further widened of the gap, especially for people with underlying mental health conditions. Such limitations, while aimed to protect the public from communicable diseases, may trigger mental health problems and ultimately mental illness (32). Texting services may help to close the emergent treatment gaps and support the public health against mental falls during the COVID-19 pandemic (33).

Despite the reported benefits of texting services in the field of mental health, it is still not clear if such effects are generalized to all mental health symptoms or conditions, and whether they are available to individuals across different age groups. In addition, the evidence regarding the cost-effectiveness of these services may need to be further explored, to understand the economic values of these programs.

Therefore, this study aimed to illustrate the role of remote delivery means, particularly text messaging programs, in the field of mental health. The literature was examined to report the service regarding their supportive, preventive and screening roles when provided to the patients with mental health disorders, caregivers, or the general public with mental health symptoms. Additionally, we explored the effectiveness, feasibility, acceptability, and economic evaluation of texting services in different contexts of mental health diagnoses or during critical times.

## Methodology

This rapid review was conducted through a systematic search in PubMed, Embase, PsycINFO, and Medline databases. The review examined the available study reviews which were run in the field of addiction and mental health and reported upon the utilization or application of text messaging programs, from the inception of the database until the 30th of September 2021.

Review of the reviews represents a comprehensive and systematic approach of analysis that could help in examining online interventions, such as texting programs. Additionally, such reviews could reliably reflect various outcomes along the desired ones, which may help in enriching the topic and supporting the results.

The search strategy was run in the electronic databases with the use of Covidence software^1^. Three reviewers/authors (RS, MA, HE) helped in reviewing the articles, and independently screened titles and abstracts and reviewed all full-text articles meeting the inclusion criteria. For each article, two reviewers were needed to independently screen the titles and abstracts of identified citations, as well as for the full text review. Discrepancies were discussed among the authors and the fourth author (VA) resolved the conflicts.

### Search Terms

The following terms were used for article extraction: Systematic review, scoping review, meta-analysis, review, analysis and; online, text, internet, message, web-based, mobile, cell phone, SMS, digital health, mobile, web, mHealth, eHealth and; depression, anxiety, mental, bipolar, psychosis, PTSD, schizophrenia, substance use disorder, alcohol, drugs.

### Selection Criteria

#### Inclusion Criteria

##### Type of the Study

Systematic review, scoping review, meta-analysis, literature review, review. Quantitative and qualitative reviews were included.

##### Intervention

Text messaging service (text message or SMS), including web and phone services. Texting services were included if they were the main intervention under study or when the technology used included text messages and controlled for in the analysis and results.

##### The Mental Health Conditions/Symptoms

Common mental health conditions, including depression, bipolar disorder, psychosis, anxiety, eating disorder, suicide (ideation/attempts), PTSD, panic disorder, substance use disorder (SUD; including different substances, alcoholism, or smoking).

##### Participants

patients with mental and non-mental health conditions, caregivers, and the general public.

##### Aim of the Used Text Service

Therapeutic, prevention, screening for or monitoring of mental health condition(s).

##### The Aim/Objective of the Study Reviews

To assess the efficacy, effectiveness, economic evaluation, adherence to the used technology, feasibility, recipients' satisfaction, usability, and health care providers acceptability (as a primary or secondary outcome)

Publications are to be in the English language.

#### Exclusion Criteria

1) Digital or online interventions that did not include text messaging services or included them but did not control for this condition while reporting the results.
2) Non-review papers.
3) Non-mental health reviews, such as general health care services.
4) Any other mental health conditions, other than those referred to in the inclusion criteria.
5) Protocols, comments, or theses.
6) Publications reporting psychological impacts of social network sites (e.g., Facebook).
7) Aim of the text messages was to: improve mental health literacy, knowledge, information about psychological conditions, relieve stigma, surveillance, training, appointment reminders, or adherence to the medications as a standalone outcome.

### Data Extraction

For eligible trials, the following data were extracted using a data extraction form:

Author name and year of publication; duration covered within the review; technology used and the details of the comparative group; targeted mental health condition(s); the aim of the review; the number of the studies included in each review; type of the included population (demographic characteristics); reported bias (e.g., selection, reporting, and publication); and the main findings/results.

## Results

### Search Results

**Figure 1:**
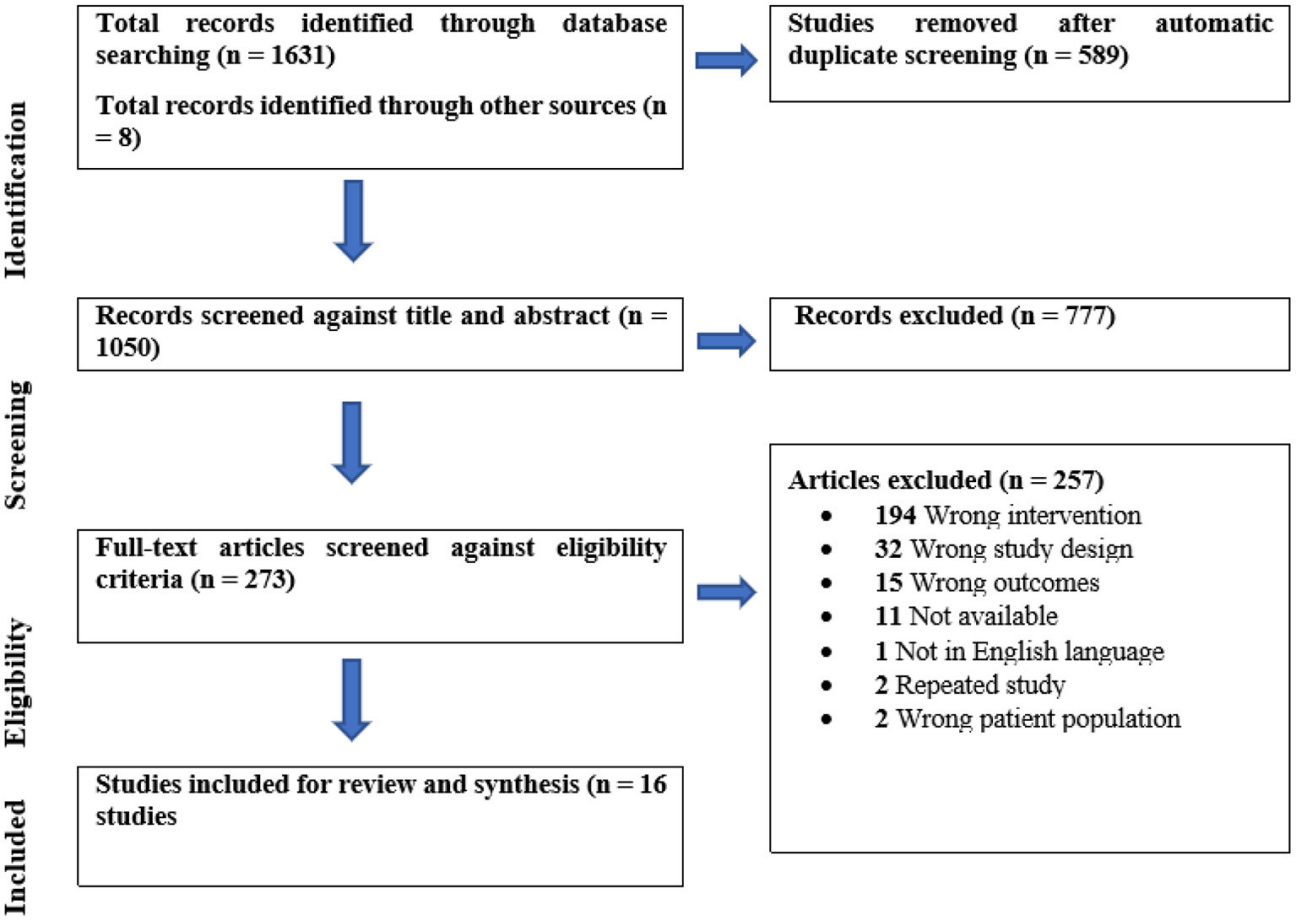
PRISMA flowchart of study inclusion process.

Through the search strategy in the electronic databases and the use of Covidence software, 1,631 articles were identified. Another eight studies were recruited manually from Google Scholar and gray Literature, yielding a total of 1,639 articles eligible for primary screening. After the removal of duplicated records, 1,050 studies were screened for title and abstract; out of them, 273 articles were eligible for full-text review. After careful review, 257 studies were excluded; the primary reason was for the wrong intervention in which the studies used different technology, such as mental health apps, iCBT, or digital technology not including texting services or did not control for texting services while reporting the results. Finally, only 16 review articles met the inclusion criteria and were included in this review Figure 1.

### Overview of the Included Reviews

Most of the included reviews were systematic reviews (*n* = 14, 87.5%); of those, five reviews were followed by meta-analysis. The rest were scoping reviews (*n* = 2, 12.5%). Publishing of the reviews started in 2014 and continued to 2021. Half of these (*n* = 8.50%) were published in the last 3 years (2019–2021). The reviews spanned the period starting from the inception of the database and ending in 2020. Refer to Table 1.

**Table 1:** Summary of studies using Web-based interventions for the mental health disorders.

**Author (Year)**	**Duration**	**Type of review**	**Technology used/ mental health condition**	**Aim of the review study**	**Number of the studies/participants' (characteristics)**	**Bias assessment**	**Outcome/results ±effect size**
Mason et al. (34)	2000–2013	Meta analysis	Text message-based intervention programs /SUD	To examine the effectiveness of text message interventions for tobacco and alcohol cessation within adolescent and young adult populations	14 articles / adolescents and/or young adults ages 12 to 29	-	-Text interventions have a positive effect on reducing substance use behaviors (Effect size = 0.25). - In general, text interventions have a positive effect on reducing substance use behaviors. - Results are discussed in the context of prevention opportunities and recommendations for future text messaging intervention research
Hutton et al. (35)	2005- January 2017	Systematic review of the literature	mHealth interventions delivered via website or mobile technology (including text messages, apps on smartphone devices, iPad and internet delivered treatment)/alcohol consumption.	To examine current evidence on the effectiveness of mHealth technology use in reducing harmful alcohol-related behaviors among young people without known alcohol addiction.	18 articles/young people (12–26 y) without known alcohol addiction, alcohol dependency, or a pre-existing condition related to alcohol	-	-The length of interventions, time carried out and follow-up was variable, with the shortest intervention being 2 to 3 h and the longest being 1year. - Ten studies reported some effectiveness related to interventions with nine reporting a reduction in alcohol consumption. - SMS messaging had the greatest efficacy among the interventions reviewed in this population. - Use of mHealth, particularly text messaging (SMS), was found to be an acceptable, affordable and effective way to deliver messages about reducing alcohol consumption to young people
Alvarez-Jimenez et al. (14)	From inception to August 2013.	Systematic review	User-led, internet or mobile-based interventions/psychosis	To systematically compile and analyze the current evidence on the acceptability, feasibility, safety and benefits of internet and mobile-based interventions for people suffering from psychosis	12 articles/No specific demographic characteristics. Participants should be diagnosed with schizophrenia-spectrum disorders using either DSM or ICD criteria.	Cochrane Collaboration ’risk of bias' tool. Only one study reported adequate randomization procedures, blinding of study-group assignments and had sufficient statistical power to detect moderate effects. For uncontrolled studies, only three measured acceptability and completion of the intervention against a priori criteria. No study included assessors blind to study purpose and/or methodology	**Regarding mobile interventions only:** -There was an associated increased number of social contacts, decreased hallucination severity, improved medication adherence, and useful medium to monitor early warning signs of relapse and possibly prevent hospital admissions in real-time ecological momentary assessments. - A tailored SMS-based intervention showed encouraging results in improving social contacts and hallucination severity. - Conversely, no significant improvements were detected in psychotic symptoms, depression and independent living skills, fostering functional outcomes through validated measures at follow-up
Bastola et al. (36)	From 2010 to 2018	Meta-analysis of RCTs and pre-post studies	Mobile phone-based text messaging/SMS/Alcohol abuse and alcoholism	To analyze the effectiveness of mobile phone-based text messages as a preventive intervention for youth and younger adult populations' problem drinking.	44 articles/College students and younger adults (<39 years).	- Cochrane “Risk of Bias” tool was used. - No results were reported.	Forest plot analysis showed reduction in binge drinking episodes in the control group without the intervention (OR = 2.45 [1.32–.53], *I*^2^ = 59%, χ^2^ = 14.64), suggesting that mobile phone-based text messaging was not effective in lowering binge drinking, with possibilities of opposite effect. - Forest plot analysis on the effect of short-term interventions using mobile phone-based text messaging on the mean drinks per occasion favored neither group (standard mean difference = 0.28 [−0.02 to 0.58], *I*^2^ = 28%, χ^2^ = 4.19), also suggesting that the text message had negligible impact on the experimental group. - Similarly, the effect of text messaging on the reduction in average standard glasses per week favored neither group (standard mean difference = −0.05 (−0.15 to 0.05), *I*^2^ = 0%, χ^2^ = 2.88), further suggesting that the text messages had a negligible impact. - Forest plot analysis on the effect of long-term interventions favored the control groups over the experimental groups (OR = 7.24 [2.71–19.31], *I*^2^ = 76%, χ^2^ = 12.41)
Berrouiguet et al. (37)	In May 2015	Systematic review of RCTs, non-RCTs and protocols	mobile phone and web-based text messaging. (Text messages could be delivered to a patient by the caregiver or vice versa). / Mental health conditions, including SUD, depression, anxiety, bipolar disorder, and schizophrenia.	To review the literature regarding the use of mobile phone text messaging in mental health care; SMS was used to promote mental health, including any type of preventive or monitoring strategy	36 articles/No special characteristics were reported.		- Text messaging was used in a wide range of mental health situations, notably substance abuse (31%), schizophrenia (22%), and affective disorders (17%). Four ways were identified in which text messages were used: reminders (14%), information (17%), supportive messages (42%), and self-monitoring procedures (42%). Applications were sometimes combined. - Growing interest in text messaging since 2006 was reported and text messages have been proposed as a health care tool in a wide spectrum of psychiatric disorders including substance abuse, schizophrenia, affective disorders, and suicide prevention. - Most papers described pilot studies, while some randomized clinical trials (RCTs). - Overall, a positive attitude toward text messages was reported.
							- RCTs reported improved treatment adherence and symptom surveillance. care services -Other positive points included an increase in appointment attendance and in satisfaction with management and health
Boland et al. (38)	From January 1980 to May 2016	Systematic review and Meta-analysis	Technology-based smoking cessation interventions (eg, mobile phone (text or apps), internet, etc.), excluding telephone counseling or VHS video), or conventional mass media campaigns / Smoking	To assess the methodological quality and effectiveness of technology-based smoking cessation interventions in disadvantaged groups.	13 studies/Disadvantaged groups (vulnerable populations or socioeconomic status or homeless persons or mental health patients or prisoners or juvenile delinquency or Indigenous/Maori/Inuit/north American Indian)	Cochrane Collaboration risk of bias tool was used. Only one study scored six or more “low risk” items and was deemed to be methodologically rigorous. Eight studies deemed to be moderately rigorous. Four studies deemed to be poor on measures of methodological rigor. The majority of studies were at low risk of bias for sequence generation, incomplete outcome data, selective reporting Approximately half of the studies were at low risk of bias for allocation concealment. None of the studies clearly reported blinding of participants and personnel, Three studies were deemed high risk of bias for incomplete outcome data; three studies were considered high risk for selective reporting; and two studies were considered high risk for other bias including uneven sample sizes and significant differences in baseline characteristics per condition	- Regarding mobile text-messaging intervention alone (only one study), a significant effect was reported after 1 month of the intervention, with higher odds of smoking cessation among intervention groups (OR 2.81, 95% CI 1.58, 4.99). - Mobile phone text-messaging, computer- and website-delivered quit support showed promise at increasing quit rates among Indigenous, psychiatric and inpatient substance use disorder patients
Cox et al. (8)	from 1992 to 18 September 2018	Systematic review and Meta-analyssi	Text messaging interventions were defined as one or more text messages with health-related content sent to a personal mobile device. The comparator had to be usual care or an attention control One-way and two-way text messaging trials were included. Trials of smartphone applications were excluded./Depression	To quantify the effects of text messaging interventions to reduce depressive symptoms Identify variables that might influence the effectiveness of the intervention	Seven studies with 1,918 participants/Adults aged ≥18 years and were identified by a healthcare provider, to minimize volunteer bias. No exclusions were made on the basis of any reported medical condition among the participants	Cochrane risk of bias tool for RCTs was used The overall rating of the quality of evidence of the effectiveness of text messaging was very low The evidence from the trials was downgraded in quality due to a high risk of selection and performance bias, heterogeneity and very wide CIs. Publication bias was not assessed due to the small number of included trials	-Borderline statistically significant reduction in depressive symptom scores between the text messaging intervention and control groups favoring intervention. -Statistically significant reductions were shown in important subgroups, such as in those using the Beck Depression Inventory (BDI) or 9-item- Patient Health Questionnaire (PHQ-9) questionnaires; where text message content was targeted at mental well-being, mood improvement and cognitive behavioral therapy information; and when the message frequency was ≥2 times per week. - Text messaging has potential to reduce depressive symptoms, however more research is required before recommendations can be made about the routine use of text messaging for the management of depressive symptoms
D'Arcey et al. (29)	From January 2000 to March 2019	Systematic review	Text message/Psychosis	To examine the clinical engagement and feasibility of SMS text messaging services in the treatment of psychosis	15 studies/Demographic restrictions were not applied		- Most studies demonstrated the positive effects of SMS text messaging on dimensions of engagement such as medication adherence, clinic attendance, and therapeutic alliance. - Particular subgroups expressed better adherence, such as patients with low baseline adherence and those living independently. Regarding feasibility outcome: five studies examined aspects of feasibility, usability, and user satisfaction. - Studies reported a good endorsement of interest (59%), wanting to continue using the intervention (47–64%), moderate-to-high ratings of effectiveness (41–87%), satisfaction (70–90%), and ease of use (80–98%); and only a small proportion endorsed harm associated with the intervention (13%). - Overall, SMS text messaging is a low-cost, practical method of improving engagement in the treatment of psychosis, although efficacy may vary by symptomology and personal characteristics
Senanayake et al. (39)	Between 2012 and 2019	A systematic review and meta-analysis of RCTs	Text messaging/Depression	To evaluate the effectiveness of text messaging interventions for the management of depression	Nine studies (945 patients: 764 adults and 181 adolescents)	Low risk of bias was expected (RCTs used), however publication bias may be expected where negative results may have not been published	- Five studies used text messaging as the only intervention, while the remaining combined text messaging with other treatment modalities such as behavioral activation or CBT. - A meta-analysis was conducted on seven selected RCTs (845 patients: 664 adults and 181 adolescents). The standardized mean reduction in depression due to text messaging interventions was 0.23 (95% confidence interval: −0.02 to 0.48). - There was evidence of heterogeneity in treatment effect between studies
Song, et al. (40)	From December 2016 to March 2017	Systematic review of RCTs	Mobile phone technologies (e.g., SMS, mHealth, interactive voice response (IVR) or app) / Unhealthy alcohol use (UHU)	To synthesize and understand the research evidence about the efficacy of mHealth interventions on various health outcomes for consumer self-control of UAU. To identify the core components to achieve these outcomes	19 studies/No specific characteristics were reported		- Over half of the SMS interventions were effective in reducing alcohol use or increasing readiness to change UAU in eight out of 12 studies (67%)
Tofighi et al. (41)	Not mentioned	Systematic review of the literature	Mobile phone messages (TM)/Drugs and alcohol dependence	To clarify the effects of TM intervention design characteristics (frequency, personalization, user-generated content, interactivity and privacy measures), patient engagement with the interventions, clinical outcomes, and potential adverse events	11 articles/No specific characteristics were reported		-Most studies demonstrated improved clinical outcomes, medication adherence and engagement with peer support groups. - TM interventions also intervened on multiple therapeutic targets, such as appointment attendance, motivation, self-efficacy, relapse prevention, and social support. - Suggestions for future research, including intervention design features, clinician contact, privacy measures, and integration of behavior change theories TM interventions offer a feasible platform to address a range of substances (i.e., alcohol, methamphetamine, heroin, and alcohol), and there is increasing evidence supporting further larger-scale studies
Fowleret al. (42)	From January 2004 to December 2015	Systematic review of the literature of RCTs	Mobile technology-based interventions (e.g., SMS, texts or apps)/Harmful use of alcohol (alcohol-dependent and non-dependent)	To summarize the current literature and determine the efficacy of mobile technology-based interventions among adult users of alcohol interventions.	Eight studies/At least 18 years of age and reported using alcohol.		-Most of the studies found positive effects of the intervention, even though the interventions themselves varied in design, length, dosage, and target population, and were pilot or preliminary in nature. - Positive effects of the intervention on several parameters included behavioral outcomes, such as a reduced average number of risky drinking days and heavy drinking days; marginally greater cumulative abstinence duration as well as a trend toward lower units of alcohol per drinking day; and a reduction in the number of drinks per drinking day, in addition to the cognitive outcomes, including increase in goal-setting willingness to reduce binge drinking days and readiness to change from beginning to follow-up. - Findings from this review highlight the promising, yet preliminary, evidence for the efficacy of mobile technology-based interventions, and in particular SMS interventions, among dependent and non-dependent adult users of alcohol.
Watson et al. (43)	From January 1999 to October 2015	Systematic review	Text messages (TM) / Mental health disorders or SUD	To characterize the impact of TM interventions on medication adherence or mental health related outcomes (such as psychiatric symptoms and social functioning) in people with mental health disorders including substance use.	Seven studies/≥18 years		- Three studies evaluated TM in patients with schizophrenia or schizoaffective disorder, two studies for chronic alcohol dependence, and two for mood disorders. - Six studies were RCTs, and one was a prospective pilot study with pre- post intervention design. TM frequency ranged from once weekly to 12 per day. - The effect of TM on medication adherence was measured in five studies; one study reporting significant improvements in the text messaging intervention group. - The effect of TM on mental health related outcomes showed significant improvements, in (71%) of the studies. on a variety of psychiatric and social functioning assessments, such as depression inventory measures. - The Brief Psychiatric Rating Scale (BPRS) and Brief Symptom Inventory (BSI) showed significant improvement, when combining text messaging and telephone contact. - Improvements in scores measuring quality of life (using the EuroQol Scale for Health-Related Quality of Life) was reported. - Patient satisfaction was assessed in one study, where patients reported greater satisfaction with the quality of care
MacDougall et al. (18)	From 2013 to 2020	Scoping review	SMS text messaging-based interventions/Mental health and addiction care	To map and categorize gaps around the use of SMS text messaging-based interventions. To identify the outcomes measured to determine effectiveness and engagement. To identify technological and clinical design features of these interventions. To identify the barriers and facilitators.	31 studies with <100 participants in each trial/Children or Adolescents <18 years		-Intervention engagement was the most common type of outcome measured (18/31), followed by changes in cognitions (16/31); and acceptability (16/31). - Interventions were typically delivered in less than 12 weeks and adolescents received 1–3 messages per week. 35% (11/31) of studies specifically indicated the intervention was for substance use or problem drinking, and 32% (10/31) focused on adolescents with depression. Bidirectional messaging was involved in 65% (20/31) of the studies. - Limited descriptions of implementation features (eg, cost, policy implications, technology performance) were reported
Dwyer et al. (44)	Up to November 2020	Scoping review	E-mental health approaches involving text-based, real-time communication with a qualified human therapist and the predictive power of language use patterns./Depression, suicidal ideation or anxiety	To map the research that has explored text-based e-mental health counseling services and studies that have used language use patterns to predict mental health status. To capture existing and emerging research within the field	70 studies/No specific characteristics were reported		-Text-based counseling is effective in treating psychological distress and depression and may be effective for treating substance abuse, reducing high-risk behaviors, and improving the subjective experiences of individuals with attention deficit hyperactivity disorder and/or autism. - When patients with depression are comfortable with online communication, the anonymity of the online therapeutic relationship may be more appealing, and online therapy is an acceptable and helpful alternative to face-to-face services. - The text-based communications can be used to predict progress accurately during treatment, and identify individuals at risk of serious mental health conditions and suicide
Berry et al. (45)	From 2005 to 2015	Systematic review	Online or mobile phone interventions/Severe mental illness (SMI): Psychosis, bipolar disorder, or personality disorder	To explore whether interventions delivered online and via mobile phones are hypothetically or actually acceptable for people with SMI. Investigate whether participant and intervention-related factors influence acceptability. Identify common participant views about acceptability from qualitative studies	49 studies/No specific characteristics were reported		-The hypothetical acceptability of online and mobile phone-delivered interventions for SMI was relatively low, while actual acceptability tended to be high, however, hypothetical acceptability was higher for interventions delivered via text message than by email

*SAD, social anxiety disorder; DSM-5, Diagnostic and Statistical Manual of Mental Disorders, fifth edition; MI, motivational interviewing; VHS, Video home system*.

### Targeted Conditions

Various mental health conditions and addiction disorders were covered in the reviews under study. Just below half of the reviews (*n* = 7, 44%) targeted the condition of SUD, including substance, alcohol or smoking (34–36, 38, 40–42). The rest included other mental health conditions: depression (*n* = 2, 12.5%) (8, 39), psychosis (*n* =2, 12.5%) (14, 29), depression, suicidal ideation or anxiety (*n* = 1, 6%) (44) or any mental health disorder (*n* = 4, 25%) (18, 37, 43, 45).

### The Intervention

More than half the review studies (9/16, 56%) focused primarily on texting-based services, while seven studies examined different online interventions that included texting services [Hutton et al. review (35), which examined text messages, apps on smartphone devices, iPad and internet delivered treatment; Alvarez Jimenez et al. review (14), which included internet or mobile-based interventions;; Berry et al. review (45), which examined Online or mobile phone interventions; Song et al. review (40), which examined mHealth, interactive voice response (IVR) or app; Boland et al. review (38), included mobile phone (text or apps)and internet services; Fowler et al. review (42), which included Mobile technology-based interventions, such as SMS, texts or apps; and Dwyer et al. review (44), which included E-mental health approaches involving text-based, real-time communication with a qualified human therapist and the predictive power of language use patterns].

### Aim of the Reviews

Most reviews aimed to assess text messaging services or mHealth interventions (controlling for text message outcomes) regarding their effectiveness (*n* = 10) (8, 34–36, 38–42, 44), acceptability, feasibility, usability, barriers, safety and benefits (*n* = 4) (14, 29, 37, 45); adherence (to medications or the intervention) or engagement (*n* = 3) (29, 41, 43); general characteristics of the service and other outcomes (*n* = 3) (18, 40, 44), with reported overlapped outcomes.

### Number of Articles/Participants Included in the Reviews

The total number of articles included in each review ranged from (*n* = 7) in two reviews (8, 43) to [*n* = 70, (44)]. The number of participants included in each review was not always reported. Only Cox, Allida et al. 2021 (8) reported having 1,918 participants for their seven studies; and Senanayake et al. 2019 (39) reported 945 participants for their nine studies.

### Participants' Characteristics

The included reviews targeted different populations; for example, four studies targeted young people, adolescents, or young adults spanning from 12–39 years (18, 34–36). Two studies (8, 40) targeted adult populations 18 years and older, and one study (39) targeted both adults and adolescents. One study (38) targeted vulnerable and socioeconomically disadvantaged populations, including homeless persons, mental health patients or prisoners, juvenile delinquency, or Indigenous /Maori /Inuit /North American Indian). The rest of the studies (*n* = 8) did not report/include any specific demographic characteristics for the participants (14, 29, 37, 40, 41, 43–45).

### Risk of Bias Assessment

Only six studies (40%) of the included reviews reported the assessment of bias among the studied articles. The Cochrane Collaboration risk of bias tool was commonly used to assess the quality and risk of bias. The results varied but overall, seem to be at a high risk of bias; for example, Alvarez-Jimenez et al. (14) demonstrated that only two studies (one RCT and one uncontrolled study) out of ten (20%) mostly met the assigned quality criteria, while no study included blind assessors to study design or purpose. Boland et al. (38) reported that only one study (1/13, 8%) was methodologically rigorous, while the rest were between moderately (8/13, 62%) and poorly rigorous (4/13, 31%). Similarly, Cox et al. (8) reported the quality of evidence of the effectiveness of text messaging among their reviewed studies was very low. The authors attributed the low quality to a high risk of selection and performance bias, heterogeneity, and the reported wide confidence intervals.

On the other hand, Senanayake et al. (39) excluded the probability of high risk of bias among their review studies, but they did not exclude the risk of publication bias that may be expected when negative results were not published.

### Outcome Results

Regarding the effectiveness of text messages in mental health conditions, of the 16 included reviews, the majority indicated that the intervention was effective either on self-reported or clinical parameters. The effect size of the intervention was reported only in one review (34). The rest of the reviews reported the effect of the intervention based on the included studies, referring to different effects reported within specific demographic subgroups or according to the duration of the service. Other outcomes were also reported. Table 1 summarizes the outcome results.

The reviews under study were classified according to the targeted clinical condition and their outcomes, as follows:

#### SUD, Including Alcohol and Smoking Cessation

Seven studies examined the effect of text messages in people with diagnosis/risk of SUD. Six of the reviews reported the positive effect of text messaging services, while only one review (36) reported negative outcomes as follows:

1. Mason et al. (34) reported positive results of texting services in their meta-analysis when used as a preventive tool for SUD in adolescents. The authors reported that some studies had a medium effect size (0.54), but overall and when including all studies, the effect size was small.
2. Hutton, Prichard et al. (35) examined the effect of mHealth interventions, including text messages, on reducing the harmful alcohol-related behaviors among young people. The authors reported that the service was effective in reducing alcohol consumption in 50% of their studies, mostly in studies that included text messages. Additionally, they found that young people liked personalized messaging that helped in an effective way to convey mHealth services.
3. Song et al. (40) examined the effect of mobile phone technologies, including SMS, on unhealthy alcohol users (UAU). The authors depicted that more than half (63%) of these interventions produced positive outcomes. In respect to SMS interventions alone, a similar percentage was reported (eight out of 12 studies; 67%).
4. Tofighi et al. (41) systematically assessed the effect of mobile texts on people with drugs and alcohol dependence. In this systematic review, the authors reported that most studies demonstrated improved clinical outcomes in terms of reduced alcohol, methamphetamine, and opioid use, and relapse prevention.
  In respect to other outcomes, the authors reported improved medication adherence, engagement with peer support groups, clinic follow-up, appointment attendance, motivation, and social support. Regarding feasibility, the authors reported that text messaging interventions are feasible platforms for addressing a range of substances, such as alcohol, heroin, methamphetamine, and alcohol.
5. Fowler et al. (42) examined the efficacy of mobile-based interventions on reducing the harmful effects of alcohol among adult alcohol users (dependent or non-dependent). In their review, the majority of the studies assessed text messages as a primary intervention (six out of eight studies; 75%). The authors reported promising results, given that most studies found positive effects of the intervention on several parameters, including behavioral and cognitive outcomes.
6. Boland et al. (38) ran a meta-analysis to examine the effectiveness of the technology-based services (phone and internet) in the context of cessation of smoking among disadvantaged vulnerable populations. The meta-analysis concluded a positive effect in favor of the intervention; where people in the intervention group were at least 1.29 times more likely to quit smoking. Regarding the mobile text-messaging intervention alone, the results were promising, where a statistically significant effect was reported after 1 month of the intervention. On the long-term analysis, although high odds were reported on 3- and 6-month follow-up among those who received the text-based intervention, the effect was not statistically significant.
7. Similar to Mason et al. (34) review (14 studies), the review of Bastola et al. (36) (19 studies) examined the text messages effect on young adults regarding alcoholism/abuse. Unlike Mason et al.'s results, Bastola et al. (36) concluded in this meta-analysis that text message-based interventions might not be effective in decreasing alcohol intake in the younger populations in the short term. Moreover, a possible opposite effect was reported in the long term.

#### Psychosis

Two reviews assessed a mobile-based intervention (14) and text message services (29) in patients with psychosis. The reviews concluded positive results related to the efficacy, engagement, and feasibility of the intervention, as follows:

1. Alvarez-Jimenez et al. (14) indicated that the treatment delivered via both the online and mobile-based interventions may show promise in improving positive psychotic symptoms among patients with schizophrenia-spectrum disorders. Moreover, mobile-based interventions may provide a useful tool to monitor early relapse signs, therefore, may prevent hospital admissions. Tailored text-based interventions were found to be particularly associated with improved symptomatology in terms of reduced hallucination severity and improved sociability but not functional outcomes.
2. D'Arcey et al. 2020 (29) reported data on text message (SMS) studied the engagement and feasibility of text messages in psychosis. The authors concluded that text messages can leverage patients' engagement in terms of medication adherence, clinic attendance, and therapeutic alliance. In terms of the measured parameters of feasibility and acceptability, overall, the service was feasible in terms of cost and practicality and was well-perceived as reported by more than half of the study participants. Overall, text messages were concluded to be safe, easy to use, and positively received.

#### Depression or Anxiety Symptoms

Three reviews focused on depression mental health condition/symptoms alone or in association with anxiety and suicidal symptoms. The results were not conclusive. Borderline significant results were reported in one review (8) and in another review when the service was accompanied by other e-services (44). The third study reported borderline significant results, however, after careful review of the data the results seem to be not statistically significant (39), as follows:

1. In the meta-analysis of Cox et al. (8), the authors demonstrated the effect of texting services on depressive symptoms among adults. The authors concluded a significant effect of the treatment delivered via text messages in reducing depressive symptom scores, however, due to the substantial heterogeneity (high inconsistency in results), the effect was described as *borderline*.
2. A similar meta-analysis of seven studies was run by Senanayake et al. 2019 (39) aimed to examine the effect of the treatment delivered via text messages on depression. The meta-analysis reported a borderline statistically-significant reduction due to text messaging (standardized mean difference of −0.23 (95% CI: −0.48 to 0.02, *p* = 0.07).
3. Dwyer et al. (44) review was the only study that examined the text message in adjunction with other e-services. The authors reported a beneficial role of e-mental health approaches on depression, suicidal ideation or anxiety, particularly when patients with depression are comfortable with online communication. In respect to text-based communications, the use of computational linguistic and modern techniques was found to help to predict progress during treatment and may ultimately identify individuals at risk.

#### Other Measured Outcomes/Mental Health Conditions

Four reviews reported on the effect of texting service on different mental health conditions. Overall, the reported outcome is seen as promising in terms of the effectiveness, satisfaction, feasibility, and acceptability of the texting service in mental health conditions, such as alcohol disorder, PTSD, SMI, suicide attempts, and anorexia/bulimia.

1. Berrouiguet et al. (37) studied the effect of text messages on different mental health conditions, including SUD. The review demonstrated various outcomes of the text messages in different mental health conditions, including SUD, schizophrenia, PTSD, suicide attempts, and anorexia/bulimia. The authors concluded that text messages may be helpful in diverse ways, additionally, they allow for inexpensive and instantaneous communication between patients and clinicians. Nevertheless, the authors reported that these interventions are at a crucial stage in their development as they present a promising opportunity for innovation; however, the risk of intrusiveness into a patient's personal space through these interventions may need to be carefully assessed.
2. Watson et al. (43) examined the effect of text messaging on mental health-related outcomes among the adult population. The authors concluded that most studies showed high satisfaction along the significant improvements in a variety of psychiatric and social functioning assessments related to mental health conditions, such as psychotic and alcohol disorders. However, regarding adherence to medications, the results were not promising. Collectively, the authors reported that these studies suggest text messaging is a promising tool to support the management of patients with mental illness.
3. MacDougall et al. (18) scoping review examined the literature for text-based services in mental health among children and adolescents. The authors reported data related to the nature, frequency, and targeted assessments of text-based services. Compared to Watson et al.'s (43) systematic review on adult populations, a smaller number and frequency of text messages were provided to the younger population, with a similar representation of the targeted clinical conditions, i.e., depression and SUD/alcohol. The authors additionally reported a high representation of engagement as a measurement outcome for the majority of the studies. This finding could not be compared to other reviews, as none reported the outcome measures in their studies.
4. Berry et al. (45) systematically examined the acceptability of remotely delivered services in severe mental illness conditions. The authors concluded that there is high *actual* acceptability of the online and mobile-based health services, while the text-based services, particularly, got higher theoretical acceptability compared to emails or phone checking. Safety and privacy were among concerns that were reported to be related to the acceptability of such interventions.

## Discussion

### Principal Findings

Our rapid review closely examined and summarized the results of 16 published reviews on the use of text-based services in the field of mental health. The reviews included in this review were mostly systematic reviews with a considerable number of meta-analysis reviews. The studies were collated between 2014 and 2021, with a majority in the latest 3 years. SUD including alcohol and smoking was the most targeted condition reviewed. This suggests that there are more reviews applied in the addiction field compared to other mental health conditions. Regarding the aim of the reviews, the effectiveness outcomes of the texting service, either as self-reported or clinically measured, represented the most assessed aim and outcome of the reviews under study (10 out of 16 studies; 63%). People of a young age were considerably represented, and the included reviews mostly reported a high risk of bias.

With respect to smoking text-based interventions, only one review reported on smoking cessation interventions via text messages (38). The review included only one study that assessed the texting service to quit smoking (46). The study showed promising results related to the intervention in young people in New Zealand. This study may pave the way to introduce further research based on evidence to support quitting smoking via supportive texting services, especially since this research was done 16 years ago and may need to be updated.

In terms of the effectiveness of the text-based services in different mental health conditions, the results are promising, particularly in psychotic disorders and SUD, including alcohol and smoking. The results related to depression ± anxiety, however, were not fully conclusive. In some studies, for example, although the effectiveness of the texting service was reported to be statistically significant, it was borderline. Additionally, the heterogeneity of the studies included in this review was generally high, thus may limit a definitive conclusion. Nevertheless, in respect to one review (8), when considering only the studies with depression as the primary outcome, which represented a majority (five out of seven studies; 71%), the sensitivity analysis reported a statistically-significant reduction in depressive symptoms with a low heterogeneity in the intervention group compared to the control group at the end of treatment (SMD, −0.30; 95% CI, −0.53 to −0.08; *I*^2^ = 23%). Similar results were obtained while considering trials using standard depression rating scales. The agreement was, therefore, hard to reach, particularly when looking at the meta-analysis run by Senanayake et al. on seven selected RCTs (39). The authors reported a borderline statistically-significant reduction in depression after the use of text messages, with evidence of heterogeneity, although the reported result does not read the same (standardized mean difference of −0.23 (95% CI: −0.48 to 0.02, *p* = 0.07), which means that the noted change may be at maximum approaching significant. For a higher quality of evidence, studies, including reviews may need to be more homogeneous and methodologically rigorous.

### Specific Findings and Comparisons

The results related to SUD may need to be interpreted cautiously, given that one review (36) reported different findings (negative) related to alcohol disorders in college students and younger adults. In this review, the authors provided a meta-analysis of 44 articles and reported odds ratios and confidence intervals for the calculated neutral to the negative effect of texting services on the study cohort. This different outcome may be explained by the specific demographic characteristics of the study cohort, as well as the clinical condition under study, particularly when the rest of the reviews, except for one study (35), examined the intervention in the adult population or did not specify the age group. Additionally, the rest of the reviews examined the SUD as a complex group rather than alcohol disorder alone as seen with Bastola et al. (36), concluding that these results may represent only the young people who have alcohol disorder. Nevertheless, Hutton, Prichard et al. (35) examined texting service in a similar cohort and mental health condition, and produced relatively positive outcomes. However, the authors reported that reduced alcohol consumption was reported only in half of their studies, and the review did not conclude that mHealth technology can definitively influence behavior change.

Regarding feasibility and acceptability, the present review reports promising results related to text messaging programs as being feasible and acceptable by the majority of the service recipients. Online supportive services were more preferred than non-support services, albeit authors reported that it is still too early to conclude that (45). A cautious approach was recommended by Berrouiguet, Baca-García et al. (37) when providing technology services, as it may carry the risk of invading the personal bubbles of patients with mental health problems. This is consistent with the reported factors that affect the acceptability of the remote health service, including safety and privacy concerns, in addition to the format of delivery and mobile technical issues (45). Similarly, D'Arcey, Collaton et al. (29) pointed out the selective effectiveness of text-based services based on baseline social characteristics. For example, patients with low baseline adherence and those who live independently reported higher effectiveness, when compared to people with high baseline adherence and those who live within a socially-supportive community. Other influences that may improve the acceptability or effectiveness were reported as receiving tailored text messages, targeted content with CBT-based information, text messages with goal-setting, and the length of registration time (8, 35). There was also a reference to the various models or frameworks applied with the included interventional texts. For example, MacDougall et al. (18) found that the social cognitive theory was the most frequently cited followed by cognitive behavioral therapy, while to a lesser extent the health belief model and normalization process theory.

Regarding the mode of delivery, given the nature of the study reviews, there was a blend of modes including both manual and automatic delivery of the messages (43), while Mason, Ola et al. (34) reported that while the majority of messages were delivered automatically, this finding was not consistently reported.

Date related to differences based on gender and to the number of messages were also reported; the high frequency of at least twice messages per week was associated with positive outcomes (8). On the other hand, a significant negative outcome was reported for male participants in the intervention arm in one study of one review (40) (one out of 19 studies, 5%), however, no change was noticed in the female participants. Variable receptivity seems built upon several socio-demographic or intervention-related factors that may, therefore, significantly influence the outcome and effectiveness of the provided service.

Digital technology, particularly text-based services, carries the promise to improve the access and quality of mental health services. Prior literature and the current report indicate high interest and acceptance of these services by the target population (18, 45). Text-based services via mobile phones offer a convenient, cost-effective for both the provider and recipient, and the service represents an accessible means of implementing population-level interventions thanks to high mobile phone ownership, ranging from 82 to 94%, and in Canada, almost 90% of residents own a smartphone and SMS text messaging is embedded in 98% of mobile phones (18, 29, 47). The mobile health services, therefore, became widely spread, albeit texting-based interventions are still at a lower level than mobile health apps (45 vs. 54%) (48).

### COVID-19 and Digital Technology

During the COVID-19 pandemic, while the need for digital health support was urged, overall, the present review did not find any review that covers specifically text messages intervention in mental health during the pandemic. In a rapid systematic review, however, the authors examined all available mental health services provided during the pandemic (49). The authors reported that such services were provided by different bodies, such as governments or academic agencies. The type of services included counseling, supervision, and psychoeducation through e-platforms, such as hotline, WeChat, online self-help psychological interventions, including CBT for depression, anxiety, and insomnia (49).

This review (49) highlighted the services provided during the pandemic, particularly the remotely delivered supports that aimed to fulfill the imposed requirements of physical distancing, and therefore were necessary to close the treatment gap emerged during the pandemic. The review illustrated the digital services, such as Videoconferencing, online programs, smartphone apps, text messaging, and e-mails have been useful communication methods for the delivery of mental health services. the authors referred to one project that has included texting services, Text4Hope (50). In Canada, a support text message (Text4Hope) program was launched to combat the psychological impact of COVID-19. The program provided 3 months of free supportive text messages for the general public in Alberta, Canada (50). Other digital and supportive services, referred to in the same review, were provided in Germany, for example, a ’Coping with Corona: Extended Psychosomatic care in Essen' (CoPE) provided psychological support for distressed individuals through four main steps: initial contact, triage and diagnosis, support via tele or video-conference, and aftercare (51). In Singapore, online psychotherapy and counseling were provided through videoconferencing platforms to psychiatric patients and the general public with distress related to COVID-19 (52).

Text messages in mental health may have their own limitations as reported in the reviews under study. This may be attributed either to the lack of research in this area or to the nature of the intervention. For example, the Alvarez et al. review (14) indicated that there was no reported support or enough data fostering functional outcomes while using online or mobile-based interventions. Similarly, coupling the text message intervention with another assistant service, such as interactive voice recognition, smartphone applications, and remote online support was suggested in the literature to support text services. However, the latter view was not supported much in the literature, given the paucity of direct comparisons against text message-only interventions. Moreover, when comparing unguided online services to guided services in study reviews, the results were usually mixed, reporting that it seems early to support this conclusion (41, 45, 53, 54).

This rapid review has several limitations. While the authors carefully examined the literature, the included studies may not represent an exhaustive list of the literature. This may be due to the nature of the search which was run on specific databases (not all), the limited search terms used, restricted inclusion criteria, and the inclusion of the English-only studies. Additionally, we did not report results for meta-analysis due to the variability of the reviews and the lack of consistent reporting of effect sizes. Therefore, the conclusions reported in this review can be viewed as future recommendations for research and policy to tailor accordingly their planned future research work. Additionally, the focus of the present research was to examine the literature specifically in regards to the texting services in mental health, thus the reviews that covered supportive mental health services provided during the COVID-19 pandemic without a particular lens on texting services were not part of this review (49). Finally, as a review of the reviews, there was a blend of supportive mental health services that were provided via texting messages, including treatment delivery, treatment support, or supportive texts, which did not specify the purpose, or the types of text services aimed to examine. The blend of remotely delivered services was also part of this review, however we solicited the data related to the texting-based service, aiming to provide a comprehensive review related to this particular intervention.

To conclude, this general review carefully examined the published reviews and provided useful information regarding the online mental health support using text-based services. The results in general were promising with evidenced impacts and reported satisfaction and acceptability of texting services in various mental health conditions, including severe mental illness. The reports related to the feasibility of these programs were positive. One study reported on cost-effectiveness, but no further cost analyses were run. Some mixed outcomes were reported in relation to some diagnoses and were attributed to the large heterogeneity and sometimes to the lack of high-quality studies. This review suggested potential areas for further research, particularly as study reviews, in different contexts, including smoking cessation, cost analyses, and to some extent reviews that carefully examine the effect of texting services in management of affective disorders. Future studies should consider SMS text messaging interventions in different contexts and during critical times, such as the COVID-19 pandemic. Texting-based services have become more convenient and appealing interventions that can fulfill the essential physical distance requirements enforced during the pandemic. As such, these remotely delivered services are capable to close the treatment gap, which has exponentially increased during the pandemic. Policy- and decision-makers, therefore, need to further support text-based services through rigorous research methodologies and with guided investments in such interventions that proved to be acceptable, cost-effective, and feasible.

## Author Contributions

RS was responsible for experimental design, data collection, data analysis and the initial draft writing. MA and HE participated in the research designing, data collection, and edited the manuscript. VA supervised the work and was involved with experimental design, concept formation, manuscript composition and manuscript edits. All authors contributed to the article and approved the submitted version.

## Funding

This work was supported by Douglas Harding Trust Fund and Alberta Innovates Award.

## Conflict of Interest

The authors declare that the research was conducted in the absence of any commercial or financial relationships that could be construed as a potential conflict of interest.

## Publisher's Note

All claims expressed in this article are solely those of the authors and do not necessarily represent those of their affiliated organizations, or those of the publisher, the editors and the reviewers. Any product that may be evaluated in this article, or claim that may be made by its manufacturer, is not guaranteed or endorsed by the publisher.

## Footnotes

^1^Covidence https://www.covidence.org/.

## References

[1] Sporinova B Manns B Tonelli M Hemmelgarn B MacMaster F Mitchell N. Association of mental health disorders with health care utilization and costs among adults with chronic disease. JAMA Network Open. (2019) 2:e199910-e. 10.1001/jamanetworkopen.2019.9910 31441939 PMC6714022

[2] WHO. World health organization & united nations development programme. mental health investment case: a guidance note. World Health Organization. Available online at: https://apps.who.int/iris/handle/10665/340246. License: CC BY-NC-SA 3.0 IGO (2021) (accessed December 5, 2021).

[3] WHO. The World Health Report 2001: Mental Disorders affect one in four people. 2001. Available online at: https://www.who.int/news/item/28-09-2001-the-world-health-report-2001-mental-disorders-affect-one-in-four-people (accessed December 6, 2021).

[4] Wittchen HU Jacobi F Rehm J Gustavsson A Svensson M Jönsson B. The size and burden of mental disorders and other disorders of the brain in Europe 2010. Eur Neuropsychopharmacol. (2011) 21:655–79. 10.1016/j.euroneuro.2011.07.018 21896369

[5] Solutions S. Top 5 Barriers to Mental Healthcare Access. (2021) Available online at: https://wwwsocialsolutionscom/blog/barriers-to-mental-healthcare-access/ (accessed in December 6, 2021).

[6] Moroz N Moroz I D'Angelo MS. Mental health services in Canada: Barriers and cost-effective solutions to increase access. Healthcare Manag Forum. (2020) 33:282–7. 10.1177/0840470420933911 32613867

[7] Statistics Canada. Health fact sheets, Mental health care needs, 2018. Available online at: https://www150.statcan.gc.ca/n1/pub/82-625-x/2019001/article/00011-eng.htm (accessed June 17, 2020).

[8] Cox KL Allida SM Hackett ML. Text messages to reduce depressive symptoms: Do they work and what makes them effective? A systematic review. Health Edu J. (2021) 80:253–71. 10.1177/0017896920959368

[9] Rodriguez-Pulido F Castillo G Hamrioui S Martin LD Vazquez-Beltran P. de la Torre-Diez I. Treatment of depression in primary care with computerized psychological therapies: systematic reviews. J Med Syst. (2020) 44:67. 10.1007/s10916-020-1543-7 32060635

[10] Wilks CR Zieve GG Lessing HK. Are Trials of computerized therapy generalizable? A multidimensional meta-analysis. Telemed J e-health : Off J Am Telemed Assoc. (2016) 22:450–7. 10.1089/tmj.2015.0129 26461235 PMC4860668

[11] Jimenez-Molina A Franco P Martinez V Martinez P Rojas G Araya R. Internet-based interventions for the prevention and treatment of mental disorders in latin america: a scoping review. Front Psychiatry. (2019) 10:664. 10.3389/fpsyt.2019.00664 31572242 PMC6753742

[12] SAMHSA. Substance Abuse and Mental Health Services Administration (SAMHSA) (2021). Telehealth for the Treatment of Serious Mental Illness and Substance Use Disorders. Available online at: https://store.samhsa.gov/sites/default/files/SAMHSA_Digital_Download/PEP21-06-02-001.pdf (accessed December 6, 2021).

[13] Gliddon E Barnes S Murray G Michalak E. Online and mobile technologies for self-management in bipolar disorder: a systematic review. Psych Rehabil J. (2017) 40:270. 10.1037/prj0000270 28594196

[14] Alvarez-Jimenez M Alcazar-Corcoles MA Gonzalez-Blanch C Bendall S McGorry PD Gleeson JF. Online, social media and mobile technologies for psychosis treatment: a systematic review on novel user-led interventions. Schizophr Res. (2014) 156:96–106. 10.1016/j.schres.2014.03.021 24746468

[15] Kumar V Sattar Y Bseiso A Khan S Rutkofsky IH. The effectiveness of internet-based cognitive behavioral therapy in treatment of psychiatric disorders. Cureus. (2017) 9:e1626. 10.7759/cureus.1626 29098136 PMC5659300

[16] Gurol-Urganci I de Jongh T Vodopivec-Jamsek V Atun R Car J. Mobile phone messaging reminders for attendance at healthcare appointments. Cochrane Database Sys Rev. (2013) 2013:Cd007458. 2 10.1002/14651858.CD007458.pub3 24310741 PMC6485985

[17] Välimäki M Hätönen H Adams CE. Mobile net: mobile telephone text messages to encourage adherence to medication and to follow up with people with psychosis: methods and protocol for a multicenter randomized controlled two-armed trial. JMIR Res Protoc. (2012) 1:e8. 10.2196/resprot.2136 23611874 PMC3626156

[18] MacDougall S Jerrott S Clark S Campbell LA Murphy A Wozney L. Text Message Interventions in Adolescent Mental Health and Addiction Services: Scoping Review. JMIR Mental Health. (2021) 8:e16508. 10.2196/16508 33416504 PMC7822725

[19] Abroms LC Boal AL Simmens SJ Mendel JA Windsor RA. A randomized trial of Text2Quit: a text messaging program for smoking cessation. Am J Prev Med. (2014) 47:242–50. 10.1016/j.amepre.2014.04.010 24913220 PMC4545234

[20] Abroms LC Johnson PR Heminger CL Van Alstyne JM Leavitt LE Schindler-Ruwisch JM. Quit4baby: results from a pilot test of a mobile smoking cessation program for pregnant women. JMIR Mhealth Uhealth. (2015) 3:e10. 10.2196/mhealth.3846 25650765 PMC4319143

[21] A Guide for Enrolling Patients and Clients in Text4baby. Available online at: https://safehealthcareforeverywoman.org/wp-content/uploads/TrainingDeckForStaffUse3_2016.pdf

[22] Evans WD Wallace Bihm J Szekely D Nielsen P Murray E Abroms L. Initial outcomes from a 4-week follow-up study of the text4baby program in the military women's population: randomized controlled trial. J Med Internet Res. (2014) 16:e131. 10.2196/jmir.3297 24846909 PMC4051747

[23] Abroms LC Lee Westmaas J Bontemps-Jones J Ramani R Mellerson J. A content analysis of popular smartphone apps for smoking cessation. Am J Prev Med. (2013) 45:732–6. 10.1016/j.amepre.2013.07.008 24237915 PMC3836190

[24] Agyapong VI Mrklas K Juhas M Omeje J Ohinmaa A Dursun SM. Cross-sectional survey evaluating Text4Mood: mobile health program to reduce psychological treatment gap in mental healthcare in Alberta through daily supportive text messages. BMC Psychiatry. (2016) 16:378. 10.1186/s12888-016-1104-2 27821096 PMC5100254

[25] Agyapong VI Ahern S McLoughlin DM Farren CK. Supportive text messaging for depression and comorbid alcohol use disorder: single-blind randomised trial. J Affect Disord. (2012) 141:168–76. 10.1016/j.jad.2012.02.040 22464008

[26] Noble JM Vuong W Surood S Urichuk L Greenshaw AJ Agyapong VIO. Text4Support mobile-based programming for individuals accessing addictions and mental health services-retroactive program analysis at baseline, 12 weeks, and 6 months. Front Psychiatry. (2021) 12:640795. 10.3389/fpsyt.2021.640795 34122173 PMC8192801

[27] Agyapong VIO McLoughlin DM Farren CK. Six-months outcomes of a randomised trial of supportive text messaging for depression and comorbid alcohol use disorder. J Affect Disord. (2013) 151:100–4. 10.1016/j.jad.2013.05.058 23800443

[28] Shalaby R Hrabok M Spurvey P Abou El-Magd RM Knox M Rude R. Recovery following peer and text messaging support after discharge from acute psychiatric care in edmonton, alberta: controlled observational study. JMIR Form Res. (2021) 5:e27137. 10.2196/27137 34477565 PMC8449293

[29] D'Arcey J Collaton J Kozloff N Voineskos AN Kidd SA Foussias G. The use of text messaging to improve clinical engagement for individuals with psychosis: systematic review. JMIR mental health. (2020) 7:e16993. 10.2196/16993 32238334 PMC7163420

[30] L. C. Mobile internet usage in Canada - statistics & facts (2021). Available online at: https://www.statista.com/topics/3529/mobile-usage-in-canada/#dossierKeyfigures (accessed December 15, 2021).

[31] ITU. International Telecommunication Union. Statistics: time series of ICT data for the world, by geographic regions and by level of development, for the following indicators (2005–2019; excel). (2019).

[32] Li S Wang Y Xue J Zhao N Zhu T. The impact of COVID-19 epidemic declaration on psychological consequences: a study on active weibo users. Int J Environ Rese Public Health. (2020) 17:3 10.3390/ijerph17062032 32204411 PMC7143846

[33] Agyapong VIO Hrabok M Vuong W Shalaby R Noble JM Gusnowski A. Changes in stress, anxiety, and depression levels of subscribers to a daily supportive text message program (Text4Hope) during the COVID-19 pandemic: cross-sectional survey study. JMIR mental health. (2020) 7:e22423. 10.2196/22423 33296330 PMC7752184

[34] Mason M Ola B Zaharakis N Zhang J. Text messaging interventions for adolescent and young adult substance use: a meta-analysis. Prevention Science. (2015) 16:181–8. 10.1007/s11121-014-0498-7 24930386

[35] Hutton A Prichard I Whitehead D Thomas S Rubin M Sloand E. mHealth interventions to reduce alcohol use in young people: a systematic review of the literature. Compr Child Adolesc Nurs. (2020) 43:171–202. 10.1080/24694193.2019.1616008 31192698 PMC7244006

[36] Bastola MM Locatis C Maisiak R Fontelo P. The effectiveness of mobile phone-based text messaging to intervene with problem drinking in youth and younger adult population: a meta-analysis. Telemed J E Health. (2020) 26:270–7. 10.1089/tmj.2018.0307 30985258 PMC7071024

[37] Berrouiguet S Baca-García E Brandt S Walter M Courtet P. Fundamentals for future mobile-health (mHealth): a systematic review of mobile phone and web-based text messaging in mental health. J Med Int Res. (2016) 18:13. 10.2196/jmir.5066 27287668 PMC4920962

[38] Boland VC Stockings EA Mattick RP McRobbie H Brown J Courtney RJ. The methodological quality and effectiveness of technology-based smoking cessation interventions for disadvantaged groups: a systematic review and meta-analysis. Nicotine Tob Res. (2018) 20:276–85. 10.1093/ntr/ntw391 28034998

[39] Senanayake B Wickramasinghe SI Chatfield MD Hansen J Edirippulige S Smith AC. Effectiveness of text messaging interventions for the management of depression: a systematic review and meta-analysis. Journal of Telemedicine & Telecare. (2019) 25:513–23. 10.1177/1357633X19875852 31631764

[40] Song T Qian S Yu P. Mobile health interventions for self-control of unhealthy alcohol use: systematic review. JMIR mHealth uHealth. (2019) 7:e10899. 10.2196/10899 30694200 PMC6371076

[41] Tofighi B Nicholson JM McNeely J Muench F Lee JD. Mobile phone messaging for illicit drug and alcohol dependence: a systematic review of the literature. Drug Alcohol Rev. (2017) 36:477–91. 10.1111/dar.12535 28474374

[42] Fowler LA Holt SL Joshi D. Mobile technology-based interventions for adult users of alcohol: a systematic review of the literature. Addict Behav. (2016) 62:25–34. 10.1016/j.addbeh.2016.06.008 27310031

[43] Watson T Simpson S Hughes C. Text messaging interventions for individuals with mental health disorders including substance use: a systematic review. Psychiatry Res. (2016) 243:255–62. 10.1016/j.psychres.2016.06.050 27423123

[44] Dwyer A de Almeida Neto A Estival D Li W Lam-Cassettari C Antoniou M. Suitability of text-based communications for the delivery of psychological therapeutic services to rural and remote communities: scoping review. JMIR Mental Health. (2021) 8:e19478. 10.2196/19478 33625373 PMC7946577

[45] Berry N Lobban F Emsley R Bucci S. Acceptability of interventions delivered online and through mobile phones for people who experience severe mental health problems: a systematic review. J Med Int Res. (2016) 18. 10.2196/jmir.5250 27245693 PMC4908305

[46] Bramley D Riddell T Whittaker R Corbett T Lin RB Wills M. Smoking cessation using mobile phone text messaging is as effective in Maori as non-Maori. N Z Med J. (2005) 118:U1494. 15937529

[47] Terry M. Text messaging in healthcare: the elephant knocking at the door. Telemed J E Health. (2008) 14:520–4. 10.1089/tmj.2008.8495 18729749

[48] Hussain-Shamsy N Shah A Vigod SN Zaheer J Seto E. Mobile health for perinatal depression and anxiety: scoping review. J Med Int Res. (2020) 22:17011. 10.2196/17011 32281939 PMC7186872

[49] Yue JL Yan W Sun YK Yuan K Su SZ Han Y. Mental health services for infectious disease outbreaks including COVID-19: a rapid systematic review. Psychol Med. (2020) 50:2498–513. 10.1017/S0033291720003888 33148347 PMC7642960

[50] Agyapong VIO Hrabok M Vuong W Gusnowski A Shalaby R Mrklas K. Closing the psychological treatment gap during the COVID-19 pandemic with a supportive text messaging program: protocol for implementation and evaluation. JMIR Res Protoc. (2020) 9(6):e19292. 4 10.2196/19292 32501805 PMC7309448

[51] Bäuerle A Skoda E-M Dörrie N Böttcher J Teufel M. Psychological support in times of COVID-19: the Essen community-based CoPE concept. J Pub Health. (2020) 42:649–50. 10.1093/pubmed/fdaa053 32307516 PMC7188143

[52] Ho CS Chee CY Ho RC. Mental health strategies to combat the psychological impact of coronavirus disease 2019 (COVID-19) beyond paranoia and panic. Ann Acad Med Singap. (2020) 49:155–60. 10.47102/annals-acadmedsg.202043 32200399

[53] Ahern E Kinsella S Semkovska M. Clinical efficacy and economic evaluation of online cognitive behavioral therapy for major depressive disorder: a systematic review and meta-analysis. Expert Rev Pharmacoecon Outcomes Res. (2018) 18:25–41. 10.1080/14737167.2018.1407245 29145746

[54] Christ C Schouten MJ Blankers M van Schaik DJ Beekman AT Wisman MA. Internet and computer-based cognitive behavioral therapy for anxiety and depression in adolescents and young adults: systematic review and meta-analysis. J Med Internet Res. (2020) 22:e17831. 10.2196/17831 32673212 PMC7547394

